# From injury to integrity: tissue engineering solutions in combined bone repair and regeneration and muscle repair

**DOI:** 10.1093/burnst/tkaf052

**Published:** 2025-07-29

**Authors:** Yesheng Jin, Yixue Huang, Jia Wang, Xinfeng Zhou, Jianan Chen, Wenge Ding, Zhihao Jia, Yong Xu

**Affiliations:** Department of Orthopaedics, The First Affiliated Hospital of Soochow University, Orthopedic Institute, MOE Key Laboratory of Geriatric Diseases and Immunology, Suzhou Medical College, Soochow University, No. 899 Pinghai Road, Suzhou 215000, Jiangsu, China; Department of Orthopaedics, The First Affiliated Hospital of Soochow University, Orthopedic Institute, MOE Key Laboratory of Geriatric Diseases and Immunology, Suzhou Medical College, Soochow University, No. 899 Pinghai Road, Suzhou 215000, Jiangsu, China; Department of Orthopaedics, The First Affiliated Hospital of Soochow University, Orthopedic Institute, MOE Key Laboratory of Geriatric Diseases and Immunology, Suzhou Medical College, Soochow University, No. 899 Pinghai Road, Suzhou 215000, Jiangsu, China; School of Life Science, Soochow University, No. 199 Ren ai Road, Suzhou 215123, Jiangsu, China; Department of Orthopaedics, The First Affiliated Hospital of Soochow University, Orthopedic Institute, MOE Key Laboratory of Geriatric Diseases and Immunology, Suzhou Medical College, Soochow University, No. 899 Pinghai Road, Suzhou 215000, Jiangsu, China; Department of Orthopaedics, The First Affiliated Hospital of Soochow University, Orthopedic Institute, MOE Key Laboratory of Geriatric Diseases and Immunology, Suzhou Medical College, Soochow University, No. 899 Pinghai Road, Suzhou 215000, Jiangsu, China; Department of Traumatic Orthopaedics, Third Affiliated Hospital of Soochow University, Soochow University, No. 185 Juqian Road, Changzhou 213003, Jiangsu, China; Cambridge-Suda Genomic Resource Center, Suzhou Medical College, Soochow University, No. 199 Ren ai Road, Suzhou 215000, Jiangsu, China; Department of Orthopaedics, The First Affiliated Hospital of Soochow University, Orthopedic Institute, MOE Key Laboratory of Geriatric Diseases and Immunology, Suzhou Medical College, Soochow University, No. 899 Pinghai Road, Suzhou 215000, Jiangsu, China

**Keywords:** Open bone injury, Musculoskeletal repair, Hydrogel material, Electrospun fiber material, Conductive material

## Abstract

The musculoskeletal system is essential for human movement. However, the increasing incidence of complex musculoskeletal injuries, which involve substantial loss of soft (muscle, skin) and hard (bone) tissues poses significant clinical challenges. Autogenous and allogeneic bone grafts are the most commonly adopted surgical methods for severe bone defects. However, severe postoperative complications, such as immune rejection and donor site necrosis, can lead to poor prognosis. Additionally, the scarcity of bone graft sources limits their application. Moreover, soft tissue injuries are often inadequately addressed in orthopedic procedures, leading to impaired muscle function and highlighting the urgent need for new strategies in integrated musculoskeletal repair. This review explores tissue engineering solutions by examining the interplay between muscle and bone physiology, elucidating their regenerative mechanisms, and evaluating innovations such as hydrogels, electrospun fibers, and conductive scaffolds for tissue repair. We advocate for integrated strategies that target the simultaneous restoration of soft and hard tissues to improve clinical outcomes.

## Highlights

- We provide a comprehensive overview of the latest developments in biomaterials and tissue engineering techniques that facilitate the regeneration of both muscle and bone tissues.
- We discuss the critical interactions between these two tissue types and how these insights can inform the design of effective treatment strategies.
- Our review highlights the challenges in current approaches and proposes future directions that could lead to significant improvements in clinical outcomes for patients with musculoskeletal injuries.

## Background

The proper functioning of bodily systems depends on the synchronized activities of diverse tissues, each performing its specific role. In the musculoskeletal system, the primary constituents include bones, tendons, skeletal muscles, and their interfaces [1]. The smooth functioning of human movement is facilitated by the coordinated operation of bones (providing supportive and lever actions), muscles (generating contraction forces), and tendons (integrating these actions) [2]. Movement is a fundamental aspect of human existence, underscoring the critical role of the bone-tendon-muscle unit in maintaining quality of life.

Musculoskeletal diseases and injuries rank as the second leading cause of global disability after mental disorders, with a 19.9% increase over the past decade [3]. Musculoskeletal disorders were projected to impose an annual economic burden of ~USD 980.1 billion in the United States [4]. Open bone injuries are an important manifestation of musculoskeletal injuries, whose incidence is on the rise due to the escalating occurrence of high-energy injuries such as traffic accidents and falls from height. Statistics show that open bone injuries account for ~10% of all musculoskeletal injuries [5, 6]. This type of injury involves soft tissue damage, including volumetric muscle loss (VML), skin defects, and vascular injury [7]. The Gustilo-Anderson classification system classifies open fractures with extensive damage to vascular networks and soft tissues as type IIIC, which is considered the most serious fracture type [5, 8, 9]. Such traumatic disorders frequently result in complex orthopedic issues, including infection, delayed healing, or nonunion, occurring at nearly twice the rate of closed fractures [10, 11]. VML is the most common complication in Gustilo-Anderson type III fractures, accounting for ~65% [12]. Patients with VML often have more than a two-fold increase in medical costs [5]. Currently, the primary clinical strategies for managing Gustilo-Anderson type III trauma include debridement, soft tissue coverage, fracture fixation, and bone regeneration, with infection prevention being key to treatment success [13]. Thorough debridement, involving the removal of necrotic and contaminated tissues, is critical during clinical management, and early wound surface coverage is conducive to controlling infection and promoting fracture healing [14]. Hence, flap transplantations that include muscles, are extensively applied in open fractures. Research indicates that combining the Masquelet technique for bone regeneration with muscle flap transplantation for soft tissue repair can increase the limb salvage success rate when treating complex open injuries in the lower extremities with segmental bone loss [15, 16]. Currently, latissimus dorsi and rectus abdominis are commonly used in autologous musculocutaneous flaps. However, they have several disadvantages, including limited source, bulky appearance, high surgical complexity, and donor area site complications [17–19]. Engineered skeletal muscle has emerged as a promising alternative to traditional muscle flaps [20]. Bone loss from open fractures often results in complications like limb shortening and fracture nonunion. Traditional autologous bone transplantation surgeries face challenges such as the scarcity of autologous bone sources and donor site complications, while allogeneic bone transplantation is beset with numerous problems, including rejection reactions [21]. Artificial bones could provide flexible options for implantation in bone defect sites. Consequently, tissue engineering strategies hold promise for both soft tissue damage and bone injuries. Nevertheless, open bone fractures are complex, encompassing both soft and bone tissue injuries. The prognosis can only be improved by considering both. However, research on integrated bone and soft tissue repair is still at an initial stage. Therefore, overcoming challenges in exploring the structure and function of the musculoskeletal system and developing methods that simultaneously fulfill the repair requirements of both tissue types by integrating hard and soft strategies is crucial for improving outcomes in open bone fracture repair.

**Figure 1:**
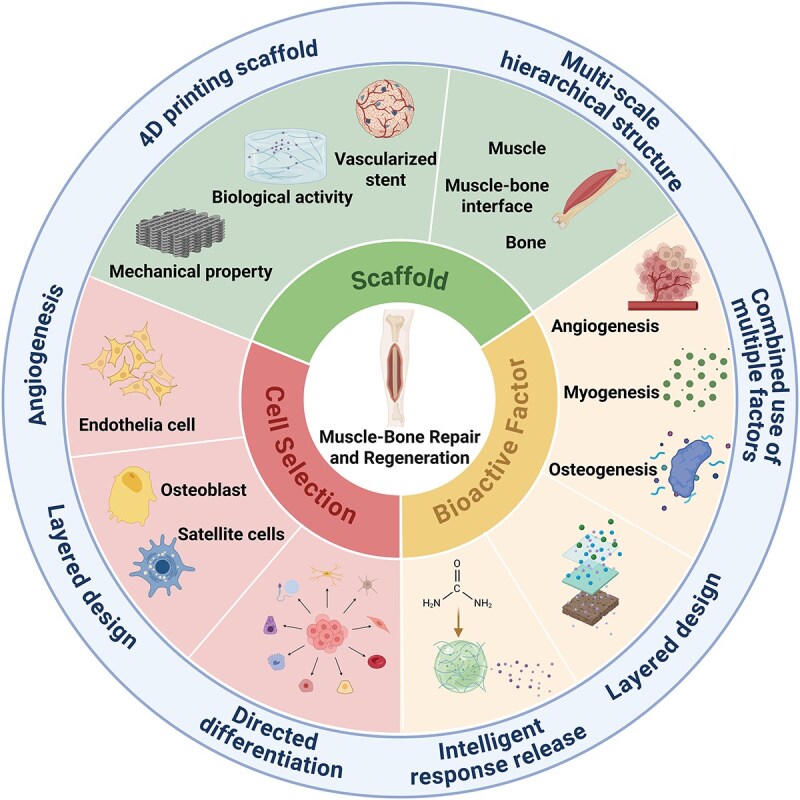
Requirements for integrated musculoskeletal repair in tissue engineering material design. The design of engineered tissue materials for integrated musculoskeletal repair should consider three aspects: Cell selection, bioactive factors, and scaffolds. Cell selection considerations include angiogenesis promotion, layered design, and directed differentiation. Bioactive factors include the combination of multiple factors, layered design, and intelligent, responsive release. Scaffolds include 4D printed and multi-scale hierarchical scaffolds. Created by BioRender. https://BioRender.com/lg5kpag

Research on isolated bone and muscle repair is relatively advanced, yet it often neglects the role of muscles in bone repair. Integrated repair of the musculoskeletal system plays a crucial role in the short-term repair of bone injuries and is indispensable in the long-term rehabilitation of sports. This review systematically examines the structure, function, and interplay among the constituents of the musculoskeletal system. It also clarifies the current understanding of bone and muscle regeneration. Moreover, advancements in tissue engineering for musculoskeletal regeneration and repair across three key domains—hydrogels, electrospun fibers, and conductive materials—are elucidated. We also discuss the prospects of tissue engineering in integrated musculoskeletal repair that integrates emerging technologies such as gradient layer design, four-dimensional (4D) bioprinting, and multi-scale biomimetic strategy (Figure 1). This comprehensive review advances the understanding of the role and significance of tissue engineering for the integrated restoration of the musculoskeletal system.

## Review

### The musculoskeletal system

The musculoskeletal system, comprising ~206 bones, 700 skeletal muscles, and associated tendons [22], presents a complex network of structural and functional interactions. A thorough understanding of these intricate dynamics is crucial for developing integrated tissue engineering materials aimed at treating open fractures effectively. The system’s role in maintaining overall health and enhancing quality of life is undeniable. Therefore, a comprehensive understanding of the musculoskeletal system is essential for developing effective engineering materials tailored for its repair and regeneration.

#### Skeletal muscle structure and function

Skeletal muscles are a crucial and essential component of the motor system. They are intricately connected to tendons and bones through collagenous connective tissue. Constituting ~45% of total body mass, skeletal muscles play critical roles in metabolism and movement, highlighting their clinical significance [23, 24]. Although skeletal muscles demonstrate remarkable regenerative potential after injury, this capacity is finite. Extensive skeletal muscle loss due to injury or surgery compromises its regenerative ability [25, 26].

**Figure 2:**
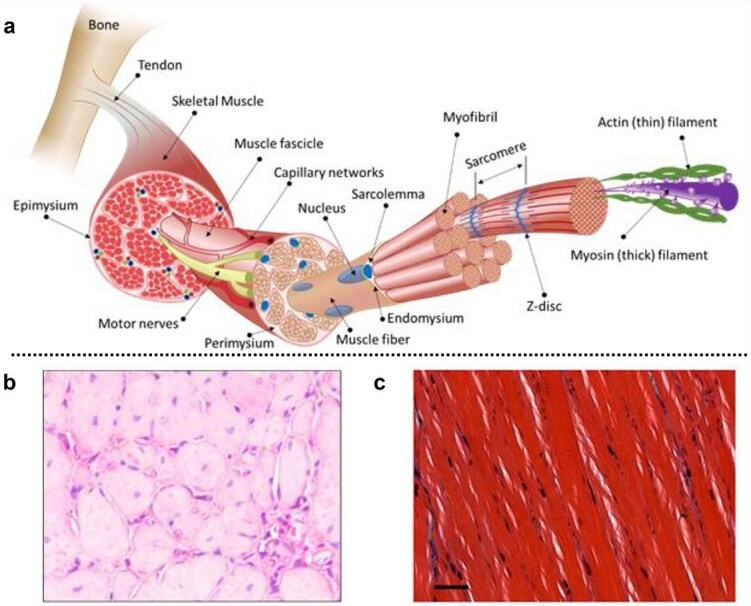
The structure of skeletal muscles. (**a**) The intricate structure of a skeletal muscle, along with the surrounding capillaries, nerves, and extracellular matrix, is organized across a hierarchy of scales ranging from millimeters to nanometers. Adapted from [23] with permission. Copyright © 2022 Wiley‐VCH GmbH., licensed under CC BY 4.0. (**b**-**c**) Cross-sectional ultrastructure of the tibialis anterior muscle, demonstrated by tissue staining. Adapted from [29, 30] with permission

The skeletal muscle structure is closely linked to its function (Figure 2). Muscle movement is facilitated by the surrounding epimysium, a smooth extracellular connective tissue that enables muscle movement relative to adjacent tissues [27]. Each muscle comprises multiple fascicles (muscle bundles), which in turn contain muscle fibers, all encased by the perimysium. The muscle fibers contain dense, well-aligned myofibrils, which are enclosed by the endomysium. Each myofibril comprises longitudinally arranged sarcomeres, the functional contractile units of skeletal muscle. Sarcomeres are composed of myosin and actin filaments. Each muscle fiber contains thousands of sarcomeres. Sarcomeres are composed of myosin and actin filaments. Each muscle fiber contains thousands of sarcomeres. Adjacent sarcomeres are connected via Z-discs that anchor α-myosin. Sarcomeres primarily consist of bundled myosin filaments and intertwined tropomyosin and actin filaments [28]. Microscopic muscle fiber contraction is initiated by the sliding motion of these filaments, which is driven by the myosin head.

#### Bone structure and function

Bones are essential for maintaining both the structural and functional aspects of life, serving as the cornerstone of various physiological activities [31]. The cellular structure of bone tissue primarily consists of osteocytes, accompanied by other specific cell types such as osteoblasts and osteoclasts. Osteoblasts, derived from bone progenitor cells, act as key mechanosensory cells that regulate bone remodeling. Osteoclasts, on the other hand, originate from macrophages and play a pivotal role in bone resorption. Together, these cells regulate the turnover and renewal of bone tissue [22].

**Figure 3:**
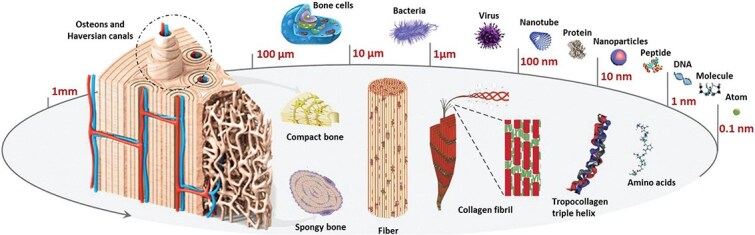
Microstructure of the bone tissue. The bone tissue microenvironment is a dynamic network of complex components such as cells, the extracellular matrix (ECM), growth factors, a controlled pH, and metabolites. Adapted from [35] with permission. Copyright © Biomater. Sci., licensed under CC BY 4.0

The bone tissue microenvironment is a dynamic network of complex components, including cells, the extracellular matrix (ECM), growth factors, a controlled pH, and metabolites (Figure 3). Bone composition is an inseparable combination of organic matrix and inorganic materials. The organic matrix, which consists predominantly of collagen (~90%), also includes non-collagenous proteins and lipids [32, 33]. Type I collagen, synthesized by osteoblasts, serves as the primary protein in the bone ECM, imparting mechanical strength to the tissue. Changes in collagen structure significantly contribute to a reduction in bone mechanical integrity, and mutations in the *COL1A1* gene, which encodes a central component of Type I collagen, are strongly associated with osteoporotic defects. The bone’s inorganic matrix consists mainly of hydroxyapatite crystals—a calcium- and phosphorus-rich neutral salt that provides structural support [34].

#### Other structures

In addition to the skeletal and muscular components, the musculoskeletal system also includes connective tissues such as tendons and ligaments. Tendon tissue comprises various cell types and a collagen-rich ECM that together optimize efficient force transmission. Within this tissue, specialized tendon cells serve as mechanosensors and actively contribute to ECM remodeling by secreting collagen [22]. Tendons are organized in a layered manner across multiple length scales to effectively withstand substantial tensile stresses. The collagenous ECM features a complex arrangement at multiple levels, contributing to the tendons’ structural sophistication. This hierarchical architecture endows tendons with exceptional tensile strength. Ligaments contain more active fibroblasts than the tendons and are composed of a larger proportion of Type III collagen and glycosaminoglycans. Their main function is to stabilize joints, limit excessive joint movement, and prevent dislocation [32].

The periosteum is a connective tissue membrane that covers the bone surface and is rich in nerves and blood vessels. Its main role is to provide nutrition to bones and participate in fracture repair and bone regeneration [36].

### Bone tissue reconstruction and healing

**Figure 4:**
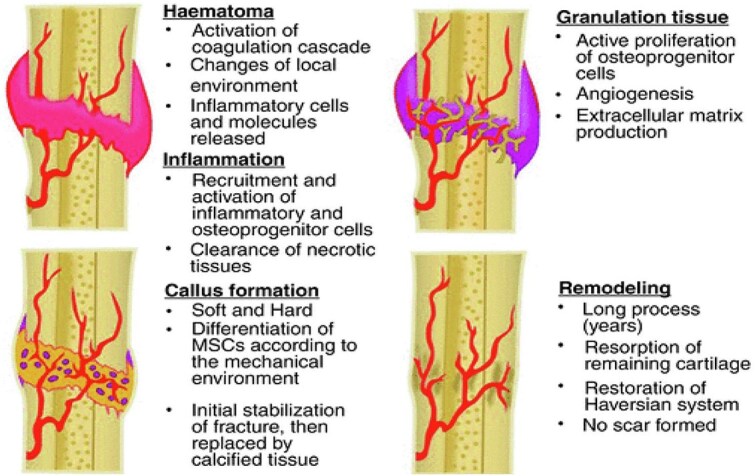
The three phases of bone regeneration. Bone regeneration progresses through three distinct pathophysiological stages: The inflammatory phase, the reparative phase, and the remodeling phase Adapted from [37] with permission

The healing process of a bone fracture typically progresses through three distinct stages: the hematoma and inflammatory phase, the repair phase (involving fibrous callus and granulation tissue formation), and the remodeling phase (characterized by osseous callus formation; Figure 4).

During the inflammatory and angiogenic stage, macrophages transition from the M1 type to the M2 type, releasing transforming growth factor-β (TGF-β) and platelet-derived growth factor (PDGF). These factors activate mesenchymal stem cells, promoting their differentiation into bone- and cartilage-forming cells. They also induce platelets to degranulate and release vascular endothelial growth factor (VEGF), which in turn promotes angiogenesis to provide nutrition for cartilage callus formation. The synergistic effect of these inflammatory cells and growth factors prepares the affected area for the repair phase, which primarily involves the transition from cartilage callus formation to bony callus formation.

Within 4–8 weeks post-injury, and before woven bone formation, bone continuity is restored through cartilage hypertrophy, matrix mineralization, and vascular reconstruction. At the regeneration site, the bone tissue transforms from a soft, connective state to a hard, supportive one. This is followed by the remodeling stage, where the mineralized callus adapts to the bone’s original shape, restoring its form and function according to mechanical requirements. This stage predominantly occurs between 8–12 weeks post-injury [38], marking the genuine transition from quantitative expansion to qualitative refinement.

At the cellular level, bone tissue repair relies on a diverse array of cells. Osteoblasts, crucial for bone formation, synthesize ECM proteins, including Type I collagen and alkaline phosphatase. These cells work together to form osteoid units, which serve as the foundational building blocks of bones. Structural support is primarily provided by Type I collagen and calcium deposits, in the form of hydroxyapatite [39].

Osteoblast differentiation depends on the presence of stem cells, predominantly bone or mesenchymal stem cells, which support bone regeneration through self-renewal, differentiation, cytokine secretion, and regulation of host tissue cells. Further elucidation is needed regarding the roles of immune microenvironment regulation and phenotypic changes of immune cells in maintaining bone homeostasis [40, 41]. Osteoclasts, which are derived from macrophages, also play a significant role and warrant special attention.

### The impact of muscular activity on bone tissue regeneration

#### Muscle tissue as an important source of stem cells for bone regeneration

Bone fracture repair and regeneration rely on the coordinated migration, proliferation, and differentiation of various stem cell types. These include, particularly, bone marrow-derived mesenchymal stem cells (BMSCs), periosteum-derived mesenchymal stem cells (PMSCs), vascular endothelial progenitor cells (VEDPs), and muscle-derived stem cells (MDSCs). Hashimoto et al. [42] demonstrated that satellite cells express myoblast (Pax7, MyoD) and osteoblast (ALP, Runx-2) markers and can spontaneously differentiate into osteoblasts. Therefore, impaired bone repair due to muscle blockage may result from a lack of MDSCs. Liu et al. [43] found that the tibial axis primarily comprises bone marrow adipose tissue and is substantially covered by muscle groups attached to its surface. The direct muscle-bone attachment minimizes separation during fractures, effectively designating this musculature as a ‘secondary periosteum’.

#### Muscle tissue integrity protects the bone regeneration process

The success of bone injury repair depends on muscle tissue integrity. Kaufman et al. [41] showed that separating the muscle layer from a fracture site with a cellulose nitrate membrane impaired bone repair. This impairment occurred because the membrane only allowed molecules of 3.5–50 kDa to pass through while blocking cells. Utvag and colleagues [44] demonstrated in 1998, in a rat fracture model, that preserving the connection between the periosteum and the muscles significantly promoted fracture healing. A subsequent study from 2002 revealed that inadequate muscle coverage was associated with delayed fracture healing in rats, indicating that muscle defects can impede early bone repair [45]. Harry et al. [46] used a mouse tibial fracture model to compare the effects of muscle and skin tissue coverage of the injury site and the vascular density in both during healing. They found that although the skin tissue had a higher vascular density throughout the healing process, the presence of muscle tissue accelerated healing.

These animal studies’ results were confirmed through clinical treatment outcomes, showing that muscle integrity was critical for long-term functional recovery and quality of life improvement in patients with musculoskeletal injuries. A meta-analysis study comparing the long-term outcomes of quadratus femoris muscle pedicle bone flap transplantation with cannulated compression screw internal fixation versus AO cannulated compression screw internal fixation for femoral neck fractures in young adults demonstrated superior results in the group receiving the combined treatment. Specifically, this group exhibited higher fracture healing rates, better hip joint function scores, reduced incidences of avascular necrosis of the femoral head, and shorter fracture healing times [47]. In a systematic review on the therapeutic effect of myocutaneous flap transplantation in adult patients with femoral neck fractures, researchers compared the therapeutic effects among femoral neck fractures in 1022 patients from 20 studies, showing similar results: myocutaneous flap transplantation has a good effect with a low incidence of avascular necrosis and nonunion [48]. Although muscle flap transplantation can improve fracture healing and long-term treatment outcomes, the healing progress is limited by infection [49], flap necrosis, and donor site dysfunction [50]. Therefore, the use of tissue engineering materials as an alternative to muscle flap transplantation becomes particularly important.

#### The periodic mechanical load generated by muscle contractions drives bone regeneration

During bone regeneration, muscle acts as a ‘dynamic regulator’. It not only directly participates by secreting various bioactive factors but also influences bone repair through mechanical stimulation. Clinically, insufficient mechanical load on bones, often caused by muscle atrophy, has been observed to lead to osteoporosis and delayed healing. This is primarily due to the mechanical load exerted by muscle contractions on bones. Muscle activity generates periodic mechanical loads on bones via tendons, activating mechanoreceptors such as integrins [51] and primary cilia [52] on osteocytes.

Studies have shown that the mechanosensitive channels Piezo1 [53] and Piezo2 are crucial force sensors for bone development and osteoblast differentiation. The absence of Piezo1, or more severely, Piezo1 and Piezo2, in mesenchymal or osteoblast progenitor cells leads to multiple spontaneous fractures in neonatal mice. This occurs because osteoblast differentiation is inhibited and bone resorption increases. Piezo1 and Piezol2 transmit fluid shear stress and ECM stiffness signals, leading to Ca^2+^ influx that stimulates calcineurin and promotes osteogenic differentiation by inducing the NFATc1-YAP1-ß-catenin signaling pathway [54].

#### Muscle tissue supplies essential cytokines for bone repair

Muscle and bone development are closely interrelated, as they are two essential components of the motor system. Movement initiation relies on muscle contraction, while bones provide the mechanical leverage for force exertion. Bioactive factors exchanged in the muscle-bone microenvironment influence the growth and development of both tissues. Hamrick, for instance, proposed that growth factors secreted by muscles can directly stimulate bone formation.

Numerous studies have documented the regulatory effects of muscle-secreted cytokines on bone growth and development. This regulation, exerted by myofactors on the bones, is intricate. Certain myofactors positively influence bone development and synthesis. For example, skeletal muscles synthesize the cytokine irisin after exercise [55]. Xue et al. [56] discovered that in mice, irisin can stimulate osteoblasts and activate the bone morphogenetic proteins (BMP)/Smad pathway via the α5 receptor to enhance bone formation. Furthermore, Hu et al. [57] indicated that irisin protects against wear-particle-induced osteolysis by alleviating oxidative stress and inhibiting RANKL production in osteoclasts. Insulin-like growth factor 1 (IGF-1), vital for tissue growth and development, is primarily synthesized in the liver but has also been shown to be secreted by extrahepatic tissues, including skeletal muscles, under the influence of growth hormone [58, 59]. Banu et al. [60] revealed that IGF-1 overexpression in mice led to increased muscle mass, cortical bone area, and bone mineral content. Conversely, certain myokines, such as interleukins, have a detrimental impact on bone formation. IL-6 is a myokine known for its role in muscle-bone interaction. A clinical study conducted among postmenopausal women with osteopenia, treated with bisphosphonates and calcitriol, found an inverse relationship between baseline serum IL-6 levels and both handgrip strength and lumbar vertebral Bone Mineral Density [61, 62].

#### Bones can have a reactive effect on skeletal muscles

The secretion of active factors by osteoblasts, osteocytes, and skeletal muscle cells is a widely observed phenomenon. Wang et al. [63] demonstrated that BMSCs facilitate skeletal muscle cell proliferation primarily through the paracrine secretion of VEGF. Osteocalcin, a hormone secreted by osteoblasts, exerts its effects via a G-protein-coupled receptor and is negatively regulated by embryonic stem cell phosphatase (Esp) tyrosine phosphatase. Studies have shown that *Esp*^−/−^ mice exhibited improved muscle quality, whereas *Ocn*  ^−/−^ mice displayed impaired muscle function [64].

### The application of tissue engineering materials in musculoskeletal repair

#### Hydrogel materials

Hydrogels are highly suitable materials for tissue engineering due to their hydrophilic polymer configuration, which enables them to retain substantial amount of water while closely mimicking the mechanical properties of human tissues [65]. Both natural and synthetic hydrogels are frequently employed as carriers for bioactive molecules, owing to their capacity for sustained release and their ability to avoid premature enzymatic degradation [66]. Hydrogels exhibit distinct applications based on their mechanical properties. For instance, high-hardness hydrogels are well-suited as scaffolds for bone regeneration, offering essential biomechanical support [67], while soft hydrogels are ideal as support for wound repair, providing minimally invasive injectability and adaptability to irregular injury sites [68, 69]. Consequently, hydrogels with adequate mechanical strength and sustained release of bioactive molecules find applications in soft tissue (e.g. muscles and skin) injury and bone regeneration and repair (Table 1).

**Table 1:** The main materials used in hydrogels for bone and muscle regeneration and repair

Hydrogel Component.	Other component(s)	Advantage(s)	Mechanical properties	Biodegradation rate	Drug release characteristics	Bone/Muscle	References
GelMA	Deferoxamine loaded poly(ε-caprolactone) (DFO@PCL) nanoparticle, manganese carbonyl (MnCO) nanosheets, and polylactide/hydroxyapatite (PLA/HA) matrix	Reduces inflammatory responses. Angiogenesis Infusible properties Inhibited osteoclast differentiation Superior osteogenic abilities	Compressive modulus: 0.90 ± 0.01 GPa Maximum compressive strength: 58.93 ± 1.46 MPa	42.1 ± 3.81% on Day 21 in PBS at 37°C	22.6 ± 0.68% within 1 day; Constant slow drug release kinetics beyond seven days	Bone	[85]
Water-soluble phosphocreatine-functionalized chitosan (CSMP)	MgO nanoparticles	Controlled Mg^2+^ release Osteogenic abilities Angiogenesis	Compressive modulus: 26.3 ± 2.0 kPa Maximum compressive strength: 195.0 ± 18.3 kPa	28 days in PBS at 37°C	Continuously release of Mg^2+^ over 28 days	Bone	[88]
Thermosensitive hydroxypropyl chitin hydrogel (HPCH)	Mesenchymal stem cells (MSCs), a 3D -printed poly (ε-caprolactone)/nano hydroxyapatite (PCL/nHA) scaffold	Mechanical properties High cell viability Stimulates macrophages to secrete growth factors and regulate inflammatory responses Angiogenesis Osteogenic abilities	Tensile modulus: 2.34 ± 0.10 MPa; Compressive modulus: 2.33 ± 0.27 MPa	No details	No details.	Bone	[89]
β-sheet RADA16-grafted glucomannan self-assembly synthesized fiber glycopolymer hydrogels (GRgel)	Non-covalently composited with 3D-printed PCL/nHA scaffold	Osteogenic differentiation of bone mesenchymal stem cells (BMSCs) Induces macrophage into M2-phonotype polarization	Comparable to native PCL/nHA scaffolds	No details	No details.	Bone	[90]
GelMA/HAMA	BMSCs; NOIS, native-constituent osteogenic inorganic salt; 3D-printed PCL framework	Mechanical strength and structural fidelity Rapid endochondral bone regeneration Regulates bone mineralization and osteoclastic differentiation, and accelerates late-stage bone maturation	Compressive modulus: 1987.0 ± 61.1 kPa	Gradually degrades by enzymatic hydrolysis	No details	Bone	[91]
Chitosan/silk fibroin (CS)	Bone morphogenetic protein 2 (BMP-2)-functionalized MgFe-LDH nanosheets; platelet-derived growth factor-BB (PDGF-BB)	Burst release of PDGF-BB and a sustained release of BMP-2 Short gelation time and low sol–gel transition temperature Enhanced mechanical properties Excellent angiogenic and osteogenic properties	LDH increases the mechanical property of the CS hydrogel	50% is retained after 36 days	A relatively fast release of PDGF-BB, accompanied by a slower sustained release of BMP-2	Bone	[79]
PEGylated poly (glycerol sebacate) (PEGS-NH2)/poly (γ-glutamic acid) (γ-PGA)	Rapamycin-loaded poly(diselenide-carbonate) nanomicelles (PSeR);	Highly sensitive ROS responsiveness Enhanced the osteogenic capacity for aged bone repair *in vitro* and *in vivo*	No details	No details	The rapamycin release rate is proportional to the H_2_O_2_ concentration	Bone	[92]
GelMA	Bisphosphonates (BPs) grafted on the GelMA microspheres; Mg^2+^	Stimulates osteoblasts and endothelial cells Restrains osteoclasts	No details	No details	Within 12 days; the BP release is fast, then slows down, and plateaus by Day 21	Bone	[93]
Guanidine-hyaluronic acid (G-HA)	Demineralized bone matrix (DBM)	Osteogenic properties Mechanical properties	Tensile modulus: Approximately 2 kPa Compressive modulus: Approximately 1.8–2 kPa	No details	No details.	Bone	[94]
Gelatin	Hydrazide alginate	Excellent mechanical properties, swelling stability, biodegradability, and biocompatibility Osteogenic properties	Tensile modulus: 0.9 MPa	Only a small amount of hydrolysis by Day 7	No details.	Bone	[95]
Sodium alginate (SA)	Montmorillonite (MMT) nanoparticles	Good mechanical stiffness and stability Promote macrophage anti-inflammatory M2 phenotype polarization Induce a favorable immunomodulatory microenvironment for the osteogenic differentiation of bone marrow stromal cells (BMSCs)	Compressive modulus: 13.76 ± 4.13 kPa	No details	Quick release in the first 3 h, slowly decreasing after 24 h, and then stabilizing	Bone	[96]
Gelatin powder	Dextran; NaIO4	Allows for strong tissue connections for wound closure Excellent hemostasis Improves muscle regeneration, reduces fibrosis, enhances vascularization, and decreases inflammation	No details	Remain intact with good stability by Day 7	No details.	Muscle	[97]
Alginate	Calcium silicate and strontium carbonate	Stimulated angiogenesis Directly promotes the expression of muscle-regulating factors (MyoG and MyoD) Inhibits M1 polarization, and promotes M2 polarization of macrophages to reduce inflammation	Tensile modulus: 210 ± 30 kPa; Maximum compressive strength: 280 ± 10 kPa	No details	Slow release over 28 days when soaked in PBS solution	Muscle	[98]
Methacrylic acid	Polyethylene glycol	Decreased the number of pro-inflammatory MHCII^+^ macrophages	No details	The *in vivo* degradation rate was dependent on the molecular weight of the PEG-AC	No details.	Muscle	[99]
Chitosan/Gelatin (CS/Gel)	Polydopamine (PDA)-modified ceramic hydroxyapatite (PDA-hydroxyapatite, PHA) and PDA-modified barium titanate (PDA-BaTiO_3_, PBT) nanoparticles	No details.	No details	No details.	1. Promotes M2 polarization of macrophages 2. Promotes osteogenesis and angiogenesis	Bone	[100]
Gelatin	A conductive poly (ethylene dioxythiophene)/polystyrene sulfonate matrix and Ca/Mn co-doped barium titanate (CMBT) nanofibers as the piezoelectric filler	No details.	Maintained their size and shape by Day 21	No details	1. The scaffold creates a promising electroactive microenvironment, which can upregulate biological responses 2. Can promote cellular osteogenic differentiation *in vitro* and neo-bone formation *in vivo*	Bone	[101]

BMPs, members of the TGF-β superfamily, are crucial for the formation of bones and cartilage. Among them, BMP-2 is particularly effective in promoting bone formation and has been approved by the United States Food and Drug Administration for orthopedic applications [70–72]. J Johnson et al. developed a functionalized Polyethylene Glycol (PEG) hydrogel to mimic collagen peptides. This hydrogel forms cross-links with MMP-sensitive peptides containing cysteine residues. It was designed to sustainably release BMP-2 and antibacterial lysozyme, simultaneously preventing *Staphylococcus aureus* infection and repairing segmental bone defects in the mouse radius [73]. An injectable hydrogel loaded with BMP-6, designed by Ke-Hung Chien et al. [74] promoted biomineralization and facilitated periodontal regeneration of maxillary-molar defects in rats. Sun et al. [75] integrated a biphasic, demineralized, decellularized allogenic bone-hydrogel scaffold with a cell-based BMP-7 delivery system to regenerate osteochondral defects. Evidently, the BMP family is essential for bone repair and restoration. Bioactive factors secreted by skeletal muscles primarily mediate the activation of BMP release. Xue et al. [56] discovered that, in mice, irisin, a myofactor produced after exercise, exerts its effects on osteoblasts by engaging α5 receptors to activate the BMP/Smad pathway and facilitate bone formation. Hydrogels delivering IGF-1 [60], nerve growth factor (NGF) [76, 77], VEGF [78], and PDGF [79] have all demonstrated effectiveness in promoting bone repair. Banu et al. [60] observed that mice overexpressing IGF-1 exhibited significant increases in muscle mass, cortical bone area, and bone mineral content. Lv et al. incorporated functionalized magnesium iron layered double hydroxide (MgFe-LDH) nanoplates into a chitosan/silk protein (CS) hydrogel containing platelet-derived growth factor BB (PDGF-BB) to develop an injectable, smart, thermoresponsive hydrogel (CSP-LB). This innovative formulation facilitates rapid PDGF-BB release while ensuring sustained BMP-2 delivery, thereby enhancing bone regeneration [79].

Actively promoting muscle repair during bone regeneration enhances the effectiveness of muscle-derived biological factors in facilitating bone injury repair. Research has shown that hydrogels doped with angiogenic factors and BMP-2 can coordinate angiogenesis and osteogenesis [80, 81]. These bioactive factors are among the primary myofactors secreted by skeletal muscle. The delivery of biological products, such as microvascular fragments [7], growth factor-rich plasma [82], and vascular-derived extracellular matrix [83], has been shown to enhance the efficacy of BMP-2 in hydrogel systems for bone regeneration. Given that angiogenesis is a critical goal for muscle injury repair, developing a delivery system that facilitates integrated bone and skeletal muscle regeneration is essential. For the first time, Ramesh Subbiah et al. [9] utilized an injectable hydrogel system to deliver two growth factors to a complex composite injury model. This approach enabled sustained release of the factors, demonstrating their significant efficacy in promoting bone healing. The regenerating bone achieved 52% of the mechanical strength of intact bone when the release of VEGF and BMP-2 was modulated; however, no synergistic effect was observed *in vitro*. By adjusting the type and dose of the delivered growth factors (e.g. delivering the angiogenic factors VEGF and PDGF), the system achieved better bone healing in the composite skeletal muscle injury than with BMP-2 alone [8]. This finding suggests that hydrogel loading systems hold potential for integrated repair of bone and skeletal muscle injuries, underscoring the necessity of refining delivery system compositions to optimize the coordination of bone and muscle injury repair.

To promote osteogenesis, hydrogels designed to closely resemble the physiological composition of natural bones have been developed. Natural bones comprise ~30% organic matrix, primarily Type I collagen. The remaining 70% is the inorganic matrix, whose main component is hydroxyapatite. Incorporation of inorganic reagents such as laponite [84], hydroxyapatite [85], and bioactive glass into hydrogels is an effective strategy to mimic natural bones and enhance the hydrogel’s osteogenic potential. Some researchers combined hydroxyapatite, β-tricalcium phosphate microspheres, hydrogels, and recombinant BMP-2 into nanoparticles, achieving greater bone volume and mass in a rat spinal fusion model than the control group.

Cellular behaviors, such as differentiation and migration, are strongly influenced by the mechanical environment. Hydrogels possessing mechanical properties akin to natural bone offer significant advantages for musculoskeletal regeneration. Given the importance of mechanical strength in hydrogels, small crosslinkers, such as glutaraldehyde, genistein, and dopamine, have been traditionally incorporated to optimize the hydrogel’s function and mechanical properties. Choi et al. developed a hydrogel by incorporating inorganic particles into a hyaluronic acid hydrogel that was conjugated with phloroglucinol to deliver BMP-2. The cooperation between the two materials significantly enhanced the hydrogel’s mechanical strength. Their hydrogel was shown to promote osteogenic differentiation *in vitro* and facilitate new bone formation in cranial defects *in vivo*. However, dense network structures in cell-encapsulated hydrogels might hinder cellular diffusion and proliferation [86]. Wei et al. proposed an alternative scheme using a BMP-2-loaded soft hydrogel with a higher rigidity than that of bone marrow. This strategy enhanced the viability and function of endogenous mesenchymal stem cells (MSCs). MSCs were observed to proliferate in soft hydrogel and then differentiate into osteoblasts in the interface between the hydrogel and the rigid surface [87]. The application of soft hydrogels in osteogenic repair offers theoretical potential for integrated bone and skeletal muscle regeneration.

#### Electrospun fiber materials

##### Electrospun fibers facilitate cell adhesion and differentiation

**Figure 5:**
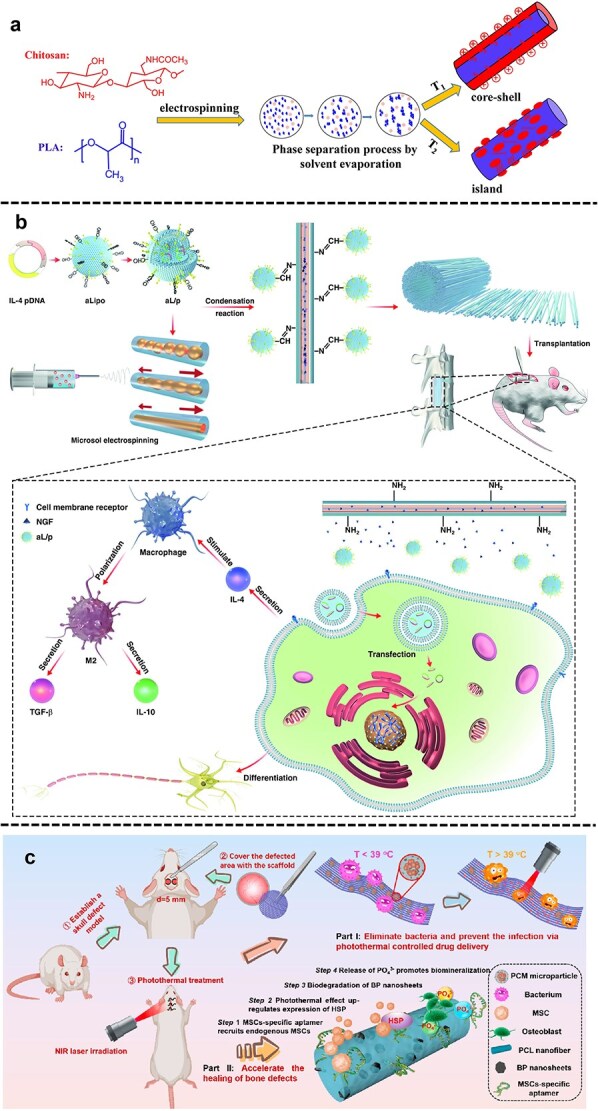
Electrospun fibers play different roles in bone and skeletal muscle injury repair. (**a**) Promote cell adhesion and differentiation. Alkaline phosphatase activity was enhanced by coating chitosan onto the fiber surface and forming ‘island-like’ protrusions that provide a more suitable interface. Adapted from [103] with permission. (**b**) The structure and mechanism of action of composite fibers, the construction of bio-inspired composite scaffolds, and their microenvironment-responsive immunomodulation and neuroregeneration effects. Adapted from [106] with permission. (**c**) A multifunctional scaffold can kill bacteria, recruit endogenous mesenchymal stem cells, and maintain a mild photothermal effect, thereby promoting bone regeneration. Adapted from [110] with permission. Copyright © 2023, American Chemical Society, licensed under CC BY 4.0

Nanofibers produced using electrospinning technology replicate characteristics of the natural ECM, providing a favorable environment for cell survival and behavior [102]. Electrospun fibrous membranes influence osteoblast proliferation and differentiation by mimicking surface roughness, dynamic compressive strain, and layering, thereby replicating the bone’s natural growth state, which is characterized by its unique layers and structure. Various rough surface textures increase the overall available surface area, enhancing cell adhesion and proliferation [37]. Some researchers isolated decalcified ECM from bones and directly converted it into nanofibers using electrospinning. Decalcified ECM, being a natural component of osteoblasts’ extracellular environment, yields fibers that closely mimic its composition and structure, providing an excellent simulation. Researchers utilized electrospinning to combine the benefits of polylactic acid (PLA) and chitosan in creating a dual-component scaffold with a core-shell and island-like structure. They demonstrated, by cultivating pre-osteoblast cells on the scaffold, that the CS-enriched outer nanofiber layer and the nanoscale rough surface effectively balanced the hydrophilicity and hydrophobicity of the material. This combination enhanced mineralization and promoted cell adhesion and growth (Figure 5a) [103].

Electrospun fiber materials are also extensively applied in soft tissue (muscle) injury repair. Electrospun materials facilitate cell adhesion, which is essential for muscle regeneration and repair. Researchers selected PCL fiber membranes of appropriate diameter and coated them with mussel-inspired poly(norepinephrine) (pNE). A combination of fibers with smaller diameters and larger specific surface areas, along with pNE-functionalized membranes, creates an ideal microenvironment for muscle cells’ viability and functionality both *in vitro* and *in vivo*. The newly formed muscle layer integrated seamlessly with the fiber membrane and surrounding tissue, achieving satisfactory mechanical strength. Electrospun fibers can be engineered to match physiological tissue stiffness and promote muscle cell proliferation. Studies have demonstrated that matrix stiffness critically influences stem cell differentiation and muscle cell proliferation. Researchers have integrated electrospun fibers with adipose stem cells to support moderate muscle reconstruction in severe VML injuries [104].

##### Electrospun fiber materials can entrap pharmaceutical drugs

Electrospinning technology can incorporate pharmaceuticals into biodegradable fibers, providing precise control over drug release. Mohammed et al. [105] developed a layered PCL/GEL co-electrospun membrane by combining substance P (SP)-loaded GEL fibers and simvastatin (SIM)-loaded PCL fibers. This material facilitated rapid SP release alongside a prolonged, controlled drug release over a month. This combination significantly enhanced cell homing, angiogenesis, and osteogenic differentiation. Black phosphorus (BP), a novel 2D crystalline material, possesses a unique layered structure and exceptional performance, including outstanding biocompatibility and high energy conversion efficiency. Using micro-sol–gel electrospinning, researchers incorporated BMP-2-modified BP nanoplatelets onto Poly-L-lactic acid (PLLA) fibers, devising a biologically driven, staged bone regeneration strategy. BMP-2 facilitates the recruitment of osteogenic precursor cells and their differentiation into mature osteoblasts, while the sustained release of phosphates from BP promotes hydroxyapatite mineralization and its 3D deposition on the fiber scaffold [72]. Xi and colleagues designed an innovative immunoregulatory approach based on pH sensitivity to enhance neural tissue repair. Their methodology involved deploying electrospun fiber scaffolds capable of microenvironmental detection in a rodent acute spinal cord trauma model. These specialized fibers exhibited rapid reactivity to the acidic conditions present in injured tissues, initiating the swift release of IL-4-encoded liposomal complexes. This mechanism effectively suppressed pro-inflammatory cytokine production while enhancing the neural differentiation potential of MSCs in controlled environments. The system employed a unique micro-sol–gel architecture with a core-shell configuration to ensure sustained delivery of NGF. Experimental results demonstrated that the immunomodulatory scaffold substantially modified immune cell population distributions, attenuated early-phase inflammatory processes, and diminished fibrotic tissue deposition. Furthermore, the intervention stimulated vascular network formation and neuronal differentiation in the lesion area, significantly improving functional outcomes (Figure 5b) [106].

Electrospun fiber-based drug systems for muscle tissue repair can be optimized by regulating drug release rates and patterns. Somiraa S. et al. [107] designed electrospun poly(ester amide) (PEA) nanofiber mats for the targeted delivery of fibroblast growth factor 9 (FGF9), a mural cell-specific factor. The delivery system’s efficacy was validated by applying FGF9/FGF2-loaded PEA mats onto the chorioallantoic membrane of chicken eggs, where enhanced angiogenesis was visualized using 3D power Doppler micro-ultrasound imaging. FGF9-loaded PEA mats were implanted on the tibialis anterior muscles of hind-limb ischemic mice, effectively delivering FGF9 to the injury site. This fostered the development of a new microvascular network with enhanced wall cell coverage within 28 days post-injury. Additionally, FGF9-treated muscles in the mice demonstrated near-normal muscle fiber size, reduced fibrosis by myofibroblasts, and improved mobility. The synergistic effect of multiple drugs is often key to single-tissue regeneration. It inspired us to consider whether an appropriate stratified drug delivery design can solve the integration problems at the soft-hard interface and the differences between osteogenesis and myogenesis.

##### Improved enhancements for electrospun fiber materials

Recent advancements have endowed electrospun materials with ‘smart’ properties, making them increasingly suitable for bone regeneration and repair [37]. Stimulus-responsive electrospun materials can adapt their volume or wettability in response to environmental changes like temperature fluctuations or infection-induced alterations. These dynamic properties enable the customization of smart material functionalities, including on-demand drug release and the regulation of cellular behavior for targeted therapeutic outcomes. For example, researchers utilized bi-electrospinning to produce a miscible nanofiber composed of polyvinyl acetate (PVAc) and PLA. Dynamic mechanical analysis identified two temperature-responsive phase transitions within the interwoven PLA/PVAc structure (Figure 5c) [108]. Some materials exhibit sensitivity to specialized environments. Liu et al. Liu et al. developed a functional 3D-printed material by integrating electrospinning with layer-by-layer 3D printing, enabling manipulation of the bone immune environment through scaffold architecture to facilitate bone regeneration. Electrospun PLLA fibers (EF) were fabricated into scaffolds, including a microfiber (M-EF) variant. Subsequently, liquid PCL was extruded through a 3D printer to form cylindrical structures (*in vivo*: 20 mm diameter, 1 mm thickness; ex vivo: 5 mm diameter, 1 mm thickness). Scaffold ink was deposited layer-by-layer onto the M-EF scaffold, forming the final 3D EF structure. Essentially, the structure was constructed by overlaying alternating layers of PCL scaffolds and PLLA fibers. This process was repeated until a six-layer PCL and five-layer PLLA 3D EF scaffold, termed ‘3D-M-EF’, was achieved. The scaffold was shown to improve the immune microenvironment and facilitate bone formation and angiogenesis *in vivo*. Furthermore, it upregulated integrin β1 expression and activated the PI3K/AKT signaling pathway, mechanisms linked to M2 polarization [109]. Zhang et al. doped BP nanosheets (BP NSs) into electrospun PCL nanofibers to endow them with excellent near-infrared (NIR) responsiveness. Then, Apt19S was modified on the scaffold surface to selectively recruit MSCs to the injured site. Under the photothermal effects of the NIR irradiation, upregulated heat shock proteins and accelerated biodegradation of the BP NSs promoted the osteogenic differentiation and biomineralization of the recruited MSCs [110]. Additional responsive materials are shown in Table 2.

**Table 2:** Various categories of responsive materials.

Response pattern	Construction structure	References
Shape	Dexamethasone (Dex)/PLMC composite nanofibers	[111]
Shape	Biodegradable poly(3-hydroxybutyrate-co-3-hydroxyvalerate) (PHBV) was incorporated to form ultrafine composite fibers through electrospinning	[112]
Shape	The amphiphilic composite comprised a poly(lactide-co-glycolide)-b-poly (ethylene glycol)-b-poly(lactide-co-glycolide) (PELGA) matrix	[113]
Shape	The generation of composite nanofibers of nanohydroxyapatite (HAp)/PLMC with diverse HAp proportions was achieved by incorporating HAp into a shape memory copolymer, PLMC, via co-electrospinning	[114]
Magnetic	Polyvinyl alcohol (PVA)/Chitosan (CS)/Fe_3_O_4_	[115]
Infection-responsive	Antimicrobial agents on the surface of the PCL nanofibrous scaffold can generate an infection-responsive guided bone regeneration/guided tissue regeneration (GBR/GTR) membrane	[116]

A significant limitation of electrospun scaffolds lies in their inherently weak mechanical properties, which restricts their clinical applicability. Nicholas et al. enhanced the scaffolds’ mechanical stiffness and strength by integrating 3D-printed meshes. Specifically, a PLA mesh was 3D-printed onto an electrospun scaffold composed of gelatin and PCL at a 60:40 ratio. The mesh was printed at strut intervals of either 0.6 or 0.8 cm. This reinforcement improved the scaffold’s mechanical properties and demonstrated biocompatibility in rat skull defect models [117]. Other researchers developed a dual-phase PCL/hydroxyapatite (3D) nanofiber scaffold using a thermally induced self-assembly technique, conferring enhanced mechanical strength for bone tissue regeneration [118]. Nafisa et al. demonstrated the fabrication of highly aligned PCL mats with collagen-like fiber architecture via near-field electrospinning (NFE). This technique promotes cell attachment and directional growth while enabling precise geometric control. The ability of NFE to construct complex, load-bearing structures paves the way for creating tissue-engineered scaffolds with heterogeneous features, such as bone dressing surfaces, and supports the development of engineered gradients for advanced tissue regeneration.

NFE enables the precise fabrication of geometrically controlled features, allowing for the integration of complex, load-bearing structures that are crucial for additive manufacturing of tissue-engineered scaffolds. This technique offers significant potential for creating heterogeneous structures, such as bone dressing surfaces, and supports the development of tissue-engineered gradients to meet specific biomechanical and biological requirements. By optimizing biomimetic ECM design, incorporating appropriate pharmaceuticals, tailoring response patterns, and improving mechanical strength, electrospun materials can more effectively promote bone and muscle tissue regeneration. The characteristics of electrospun fiber materials highlight their considerable potential for integrated musculoskeletal repair.

#### Conductive materials

Bioelectricity is a fundamental component of the body’s physiological processes. At the cellular level, most mammalian cells exhibit a stable and long-term voltage potential across their membranes, known as the membrane potential (Vm). This potential typically ranges from ~−10 to −90 mV, depending on the cell type. The membrane potential is critical in regulating processes such as signal transduction, energy conversion, and intercellular communication. Action potentials determine cellular excitability, particularly in neurons and muscle cells. Bioelectrical signals are essential for nerve signal transmission, synchronized cardiomyocyte contraction, and skeletal muscle contraction. Endogenous piezoelectricity, observed in biomacromolecules with low symmetry, is thought to be intricately linked to tissue growth and remodeling, particularly bone regeneration [119]. Electrophysiology fosters bone regeneration and repair by creating beneficial electrical microenvironments. Electrical stimulation directly influences bone-related cells, including osteoblasts, osteocytes, and osteoclasts. These electrical signals regulate the metabolism and proliferation of these cells, thereby enhancing bone regeneration. Furthermore, electrophysiological stimulation enhances vascular dilation, permeability, and increases blood flow at bone defect sites—critical factors for angiogenesis. Electrophysiology plays an equally vital role in skeletal muscle signal transduction. Its integration with bone regeneration presents novel therapeutic possibilities for combined bone and muscle repair [100].

**Figure 6:**
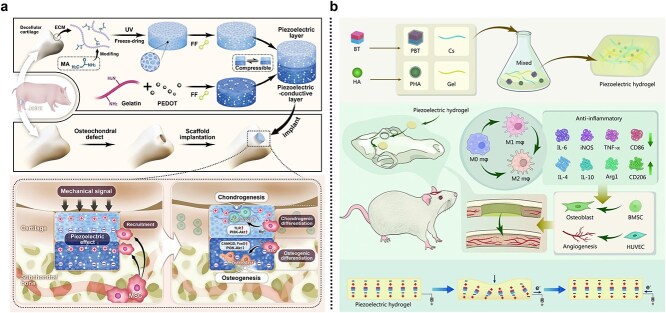
Application of conductive materials in bone and skeletal muscle injury repair. (**a**) Schematic diagram of a biodegradable piezoelectric conductive scaffold for bone and cartilage defect repair and regeneration. Adapted from [120] with permission. Copyright © 2022 Wiley‐VCH GmbH., licensed under CC BY 4.0. (**b**) Design strategy and chemical illustration of piezoelectric CG/PHA/PBT hydrogel for musculoskeletal regeneration. Adapted from [100] with permission

The remarkable regenerative capacity of bones is significantly influenced by endogenous electrical signals inherent to bone tissues. Under mechanical stress, compressed bone regions become electronegative, leading to bone resorption, while stretched regions become electropositive, promoting bone formation. Additionally, tissue damage induces the damage potential, which generates an endogenous current that aids wound healing. Current designs of piezoelectric materials for bone regeneration and repair primarily focus on promoting osteogenesis by osteoblasts and inhibiting bone resorption by osteoclasts. Several studies have proposed conductive scaffolds composed of piezoelectric cartilage-decellularized ECM and piezoelectric conductive modified gelatin. The scaffold’s piezoelectricity is induced by modifying the pore surface with diphenylalanine, while its conductive properties are achieved by incorporating poly(3,4-ethylenedioxythiophene). *In vitro* experiments have shown that BMSCs undergo biphasic division during differentiation. The positive charges accumulated on the upper layer of the scaffold attract BMSCs, facilitating their upward migration and chondrogenic differentiation. Meanwhile, the negative charges on the lower layer induce osteogenic differentiation of BMSCs (Figure 6a) [120].

Wu et al. developed an innovative piezoelectric hydrogel scaffold for bone tissue engineering by integrating PDA-modified piezoelectric barium titanate (PDA-BaTiO_3_, or PBT) and hydroxyapatite nanoparticles into a chitosan/gelatin hydrogel. This chitosan/gelatin/hydroxyapatite/barium titanate (BTO)-based piezoelectric hydrogel scaffold regulates macrophage polarization, enhances osteogenic differentiation and angiogenesis, and accelerates bone regeneration and remodeling (Figure 6b) [100].

Polyetheretherketone (PEEK) exhibits favorable mechanical adaptability and is widely utilized in repairing maxillofacial defects. However, its inherent osteogenic properties necessitate improvement. Li et al. leveraged the piezoelectric effect of BTO, utilizing low-intensity pulsed ultrasound (LIPUS) activation and PDA mediation, to engineer an ultrasound-responsive PEEK composite (PDA@BTO-modified SPEEK, PBSP) for enhancing maxillofacial bone defect restoration. PBSP exhibits enhanced hydrophilicity, which promotes cell viability and adhesion. Additionally, by harnessing the piezoelectric properties of BTO, PBSP achieves a piezoelectric coefficient comparable to that of cortical bone. Notably, PBSP generates stable electrical signals when stimulated by LIPUS, effectively accelerating osteogenic differentiation of osteoblasts [121].

Wu et al. also introduced piezoelectric materials and silver nanoparticles into hydrogels to fabricate a dual-layered piezoelectric hydrogel with antibacterial properties and enhanced mechanical responsiveness, promoting effective bone and cartilage injury repair. The introduction of sulfur (S) atoms into functional piezoelectric BTO to create sulfur-doped BTO (SDBTO) significantly enhances its piezoelectric catalytic performance, enabling effective therapeutic effects on bone defects infected with *S. aureus* and promoting osteogenesis. SDBTO-1 is activated by ultrasound to generate piezoelectric signals that effectively promote BMSCs’ osteogenic differentiation. This differentiation is achieved by upregulating the TGF-β signaling pathway, greatly improving the osteogenic microenvironment and facilitating musculoskeletal regeneration.

The application of piezoelectric materials in musculoskeletal regeneration is gradually becoming a new research hotspot. Studies have suggested that various treatments with piezoelectric materials in animal models of VML injury can enhance muscle function compared to untreated animals. These findings indicate that the combination of cell-free biomaterials and cellular components represents the most effective approach to date for improving functional recovery following VML injury. Regenerative therapies incorporating piezoelectric materials may greatly facilitate the recovery of musculoskeletal function. Researchers from Xi’an Jiaotong University discovered that nano-gold-silver alloy materials can significantly promote skeletal muscle regeneration. Furthermore, their study revealed that nano-alloy particles regulated skeletal muscle differentiation by activating the p38α MAPK signaling pathway. It was demonstrated *in vivo* that these materials substantially enhance muscle fiber and capillary formation within the defect area, leading to successful skeletal muscle regeneration [122].

Although the application of piezoelectric materials in musculoskeletal regeneration is still in the research phase, these preliminary results suggest their potential as effective strategies for treating large-volume skeletal muscle loss. Future research should focus on optimizing material performance and exploring their potential for clinical applications.

Researchers from Tomsk Polytechnic University, Russia, have developed a novel material that promotes the regeneration of bone, skin, and nerve tissues through surface treatment with dinitrogen chemicals and the promotion of osteoblast growth on piezoelectric polyhydroxybutyrate scaffolds. Moreover, the synergistic use of piezoelectric materials in both soft and hard tissues provides an opportunity for the integrated repair of bones and skeletal muscles. Some researchers have constructed Janus nanofiber scaffolds with piezoelectric properties and bionic collagen fiber arrangement to promote the simultaneous healing of tendons and bones. Double-layer fibers of polylactic acid/zinc oxide and polylactic acid/bismuth titanate were prepared by electrospinning, with their direction controlled by the spinning speed. The bionic fiber arrangement endows the scaffold with significant tensile and suture-retention strength, meeting the mechanical requirements for tendon-bone healing. Integrating ZnO and BTO nanoparticles into the PLLA matrix produced excellent piezoelectric responses, respectively inducing tendon and osteogenic differentiation [36].

#### Future perspective

Our comprehensive summary of the basic structure of bones and skeletal muscles, their repair processes, and existing tissue engineering materials revealed common challenges in current integrated muscle-bone repair attempts:

1. Repair difficulties due to tissue heterogeneity: Muscles and bones differ significantly in structure, mechanical properties, and regeneration mechanisms. Muscles require elasticity and contractility, whereas bones need high rigidity and load-bearing capacity. Integrated repair demands simultaneously meeting the unique requirements of both tissue types—a challenge for current materials.
2. Challenges in simulating the muscle-bone interface with current tissue engineering materials: (i) Artificial materials struggle to replicate the gradual change in mechanical properties found at the natural muscle-bone interface; (ii) Scaffolds often lack the necessary factors and seed cells to simulate natural biological activity to simultaneously promote the regeneration of both muscle and bone tissues.

These material design limitations—particularly regarding mechanical property matching at the soft-hard tissue interface, regulating biomaterial degradation rates, and biocompatibility issues—pose considerable challenges for preclinical studies and their subsequent clinical translation.

Therefore, future tissue engineering material designs for integrated muscle and bone repair should focus on the following aspects: (a) develop carrier materials with a gradient layering design to facilitate effective mechanical transduction at the muscle-bone interface; (b) employ a combined and coordinated approach using cytokines and seed cells to support the regeneration of various tissues at different interfaces; (c) utilize modern programming to develop precise digital designs that promote the directional differentiation of stem cells, thereby addressing the tissue heterogeneity at the muscle-bone interface.

Based on existing technologies, we propose the following prospects for the three elements of tissue engineering.

##### Cells

Seed cell selection is crucial for integrated musculoskeletal repair. BMSCs, muscle-derived progenitor cells (including muscle progenitor cells and satellite cells), and blood vessel-derived endothelial cells all play significant roles in this repair process. The layered arrangement of cells is conducive to their integrating at the musculoskeletal interface.

A team from the Shanghai Institute of Ceramics utilized multi-cellular 3D printing technology to layer tendon stem/progenitor cells and BMSCs. Together with manganese silicate nanoparticles, they constructed a gradient scaffold mimicking the tendon-bone interface. This design promoted bidirectional osteogenic and myogenic differentiation, regulated M2-type macrophage polarization by releasing Mn^2+^, inhibited inflammation, and enhanced tissue integration [123].

Other researchers have devised a minimally invasive treatment involving injecting a composite micro-tissue containing C2C12 poly (lactic-co-glycolic acid) porous microspheres and HUVEC poly (ethylene glycol) hollow microrods (PEG-HMs). These injections exhibited significant myogenic capacity, promoted the viability and function of skeletal muscle stem cells, rebuilt the vascular network, and facilitated ECM establishment [112].

Another way to address this problem is to control directed stem cell differentiation at various sites, made possible by digital light processing bioprinting technology. This process precisely replicates the complex microstructure of bone tissue. Subsequent long-term directed cultivation *in vivo* and *in vitro* leads to differentiation into various bone marrow cells, including blood, immune, and vascular endothelial cells, chondrocytes, osteoblasts, adipocytes, and osteoclasts. The resulting product has good mechanical properties (Young’s modulus in the MPa range, similar to cancellous bone), topological structure similar to bone (a loose, porous micro-nano structure), and large size (centimeter level), making it a functional bone-like organ. The organ-like scaffold showed strong repair ability in bone defect models [124].

The main issue addressed by seed cell selection is the differences in cell types and their differentiation directions at the muscle, interface, and bone surfaces. The layered design of selected seed cells effectively simulates these differences at the muscle-bone interface. Future developments in muscle-bone injury repair should focus on the cell-oriented differentiation capabilities provided by digital programming.

##### Scaffolds

The inherent differences in the natural configurations of bone and muscle tissues underscore the critical importance of designing scaffold materials that meet the distinct mechanical requirements of both tissue types. Specifically, scaffolds must provide sufficient hardness for bone tissue development and flexibility for muscle tissue regeneration, necessitating effective integration of both properties at the interface. Bionic 3D printing provides a promising solution for creating gradient scaffolds. 3D bioprinting has emerged as a multifunctional biomanufacturing technology capable of precisely controlling the composition, spatial distribution, and structure of the produced bionic tissue structures. This allows for effective mimicry of the characteristics and biological functions of the target tissues and organs. Therefore, 3D bioprinting could effectively replicate the complex structures of natural muscle and bone tissues. However, 3D printing design for musculoskeletal integration also has notable limitations. First, 3D printing is restricted to producing scaffolds with fixed geometric shapes, which cannot adapt to the dynamic changes during tissue regeneration. Second, the release of growth factors in 3D-printed constructs is predominantly driven by passive diffusion, making it challenging to achieve spatially and temporally controlled release. Additionally, 3D printing still struggles to mimic the dynamic mechanical properties of natural tissues, particularly the mechanical stress transduction at the tendon-bone interface [125].

Researchers also utilized extrusion 3D printing technology to fabricate nano-polylactic-co-tris(methylcarbonate) (PLMC) scaffolds that incorporated PDA nanoparticles as the nano-engineering material. These 3D-printed scaffolds demonstrated excellent (>99%) and rapid (<30 seconds) shape recovery under NIR irradiation. Compared with pure PLMC, the PLMC-PDA composite significantly enhanced alkaline phosphatase (ALP) secretion and mineral deposition, presenting a markedly higher *in vitro* osteogenic potential [126].

4D bioprinting, a recently developed technology based on 3D bioprinting, addresses these challenges and simulates the dynamic changes of native tissues more accurately [127]. 4D printing can dynamically align with the evolution of tissue interfaces, such as the stiffness gradient changes during bone defect healing. Some researchers employed 4D printing technology to incorporate BP nanosheets and osteogenic peptides into β-tricalcium phosphate/polylactic-co-tris(methylcarbonate) nanocomposite materials, constructing photothermal-responsive, shape-memory scaffolds for bone tissue engineering. The scaffold temperature rapidly increased to 45°C upon exposure to NIR irradiation, enabling reconfiguration of its shape for easier implantation and precise fitting of irregular bone defects. Once implanted, the scaffold temperature promptly decreased to 37°C, exhibiting mechanical properties comparable to human cancellous bone. Bone formation at the defect site was improved through the controlled release of peptides from the scaffold [128].

Researchers successfully utilized 4D printing to print various bionic muscle models, derived from 4D nanostructures, that possess adjustable mechanical properties. These structures responded to electrical signals and demonstrated good biocompatibility [129]. 4D printing can also achieve a responsive release of biological factors and facilitate multi-stage responsive design [130, 131]. A gradient mechanical design of 4D printed scaffolds could effectively address the unique mechanical stress transmission at the muscle-bone interface, a challenge that traditional scaffolds have failed to achieve. Electroactive polymers (EAPs) are a new type of smart material that can undergo shape changes when stimulated by an electric field. These polymers possess special electrical and mechanical advantages. Compared with traditional piezoelectric materials, EAPs are lightweight, have high driving efficiency, and have good seismic performance. Artificial muscle actuators based on combined EAPs and 4D printing have strong strain performance and are one of the most promising biomimetic materials [101].

4D printed engineered blood vessels are also of great significance for integrated musculoskeletal repair. Rana et al. developed programmable bioinks to promote a dynamic presentation of VEGF to guide vascular morphogenesis in 3D bio-printed constructs. Compared with untreated samples, treated samples showed a 1.35-times higher vascular density, 1.54-times higher branch density, and 2.19-times higher average vascular length [132].

The focus of improved scaffolds still lies in integrating soft and hard tissues at their interface and the issue of mechanical force transmission. 4D-printed and multi-scale biomimetic gradient scaffolds can partially resolve these challenges.

##### Bioactive factors

Biologically active and angiogenic factors are widely regarded as effective treatments for promoting tissue angiogenesis. Factors such as VEGF are continuously secreted in ischemic muscle tissue, promoting angiogenesis and increasing blood vessel density at the injury site. The strategic combination of BMP-2 and PDGF-BB is essential for musculoskeletal regeneration and repair. Ma Y et al. [133] constructed therapeutic small extracellular vesicles (t-sEVs) loaded with VEGF-A and BMP-2. When encapsulated in a custom injectable polyethylene glycolized polyglycerol sebacate acrylate (PEG-A) hydrogel, these t-sEVs demonstrated excellent bone regeneration of critical-sized defects in rat femurs. Fitzpatrick V et al. [134] integrated 3D-printed macroporous scaffolds with BMP-2, VEGF, and NGF. They found that this combination could promote the regeneration of both soft and hard tissues, highlighting the importance of using multiple factors in integrated muscle and bone repair. Different growth factors exert varying biological effects at different interfaces of the musculoskeletal system. Therefore, the stratified design of biological factors plays a crucial role in directing the differentiation of stem cells at these various interfaces. Utilizing the optimized decellularization vacuum aspiration device method, researchers prepared a book-shaped decellularized interstitial matrix (O-BDEM). Subsequently, they fused collagen-binding peptides (CBP) to the N-terminus of BMP-2, TGF-β3, and Growth Differentiation Factor-7 to create recombinant growth factors (CBP-GFs). These were then attached to O-BDEM to fabricate a novel scaffold (CBP-GFs/O-BDEM) with superior biological activity for inducing stem cells to simultaneously enter the osteogenic, chondrogenic, and tendonogenic lineages [135].

The developments in bioactive factors aim to address the requirements for different factors at various interfaces. Layered design and the combined use of several factors help address this problem, but further research in this direction is needed.

## Conclusions

As our understanding of the interaction between bone and muscle advances, we have witnessed a growing emphasis on integrative bone and muscle tissue engineering. While a solid foundation exists in organ engineering materials for musculoskeletal tissue regeneration, the development of integrated bone-muscle engineering materials remains in the exploratory phase. Several promising designs have made progress toward integrated repair; however, persistent challenges require further refinement. This review aimed to describe the structures and functions of bones, muscles, and their interactions. It highlighted the indispensable significance of both tissues in musculoskeletal regeneration and repair. Furthermore, it presented a systematic overview of three key tissue engineering material categories used in muscle and bone regeneration. Based on the reviewed data, we proposed new directions for designing novel tissue engineering materials that could facilitate integrative bone-muscle regeneration and repair.
